# Laquinimod rescues striatal, cortical and white matter pathology and results in modest behavioural improvements in the YAC128 model of Huntington disease

**DOI:** 10.1038/srep31652

**Published:** 2016-08-16

**Authors:** Marta Garcia-Miralles, Xin Hong, Liang Juin Tan, Nicholas S. Caron, Yihui Huang, Xuan Vinh To, Rachel Yanping Lin, Sonia Franciosi, Spyros Papapetropoulos, Liat Hayardeny, Michael R. Hayden, Kai-Hsiang Chuang, Mahmoud A. Pouladi

**Affiliations:** 1 Translational Laboratory in Genetic Medicine, Agency for Science, Technology and Research (A^*^STAR), 8A Biomedical Grove, Immunos, Level 5, 138648, Singapore; 2 Singapore Bioimaging Consortium, Agency for Science, Technology and Research, Singapore, Singapore; 3 Centre for Molecular Medicine and Therapeutics, Child and Family Research Institute, University of British Columbia, Vancouver, BC, V5Z 4H4, Canada; 4 Teva Pharmaceutical Industries Ltd., 5 Basel St., Petach Tikva, 4951033, Israel; 5 Department of Medicine, Yong Loo Lin School of Medicine, National University of Singapore, 117597, Singapore

## Abstract

Increasing evidence supports a role for abnormal immune activation and inflammatory responses in Huntington disease (HD). In this study, we evaluated the therapeutic potential of laquinimod (1 and 10 mg/kg), a novel immunomodulatory agent shown to be protective in a number of neuroinflammatory conditions, in the YAC128 mouse model of HD. Treatment with laquinimod for 6 months rescued atrophy in the striatum, in certain cortical regions, and in the corpus callosum of YAC128 HD mice. Diffusion tensor imaging showed that white matter microstructural abnormalities in the posterior corpus callosum were improved following treatment with low dose (1 mg/kg) laquinimod, and were paralleled by reduced levels of interleukin-6 in the periphery of YAC128 HD mice. Functionally, treatment with laquinimod (1 and 10 mg/kg) led to modest improvements in motor function and in depressive-like behaviour. Taken together, these results suggest that laquinimod may improve some features of pathology in HD, and provides support for the role of immune activation in the pathogenesis of HD.

Huntington disease (HD) is an inherited, progressive neurodegenerative disorder caused by a dominant mutation in huntingtin (*HTT*), a ubiquitously expressed gene^1^. The clinical phenotype of HD includes psychiatric disturbances such as depression and psychosis, cognitive deficits, and impairment of motor function with abnormal voluntary (gait, balance, hand movements) and involuntary movements (chorea and dystonia)^1^. Neuropathologically, the disease is characterised by striking atrophy of the striatum and thinning of the cortex, which is accompanied by early and progressive white matter loss^1^. Volumetric MRI changes in HD include pronounced caudate atrophy accompanied by putaminal and whole brain atrophy early in the disease course^2^. Despite the significant unmet need and intensive efforts to develop therapies over the past three decades, no effective treatment to reverse, stop or slow the progression of HD has been identified^3^.

Several disease mechanisms have been implicated in HD, including aberrant synaptic signalling, transcriptional dysregulation, altered proteolysis, impaired intracellular trafficking, and loss of neurotrophic support^4^. Increasing evidence also supports a role for abnormal immune activation and inflammatory responses in HD^5^,^6^,^7^,^8^. Significant accumulation of microglia, the CNS-resident macrophage, is seen in regions with neuronal loss proportionate to the stage of disease and the extent of atrophy^9^. Positron emission tomography also reveals widespread microglial activation *in vivo* in pre-manifest gene carriers, which correlates with striatal neuronal dysfunction and predicts disease onset^10^,^11^,^12^. Levels of proinflammatory immune factors are elevated in cerebrospinal fluid and plasma samples from patients with HD years before disease onset and correlate with microglial activation and disease progression^13^,^14^,^15^. These observations indicate that pathological immune activation is an early event that may contribute to disease processes in HD.

While abnormal immune activation in HD may, in part, reflect a reactive response to mutant HTT-induced cellular degeneration, the available evidence also supports cell-autonomous effects in immune cells. Indeed, HTT is expressed in B cells, T cells, and monocytes^14^,^16^,^17^. *Ex vivo* stimulation of monocytes and macrophages isolated from HD patients results in secretion of elevated levels of proinflammatory factors, an effect also seen in macrophages and microglia isolated from rodent models of HD^14^,^18^. Monocytes and macrophages from HD patients and rodent models also show impaired chemotaxis in response to stimuli *in vitro*, and HD microglia exhibit a reduced velocity of migratory processes and a delayed response in a rodent model of acute brain injury^19^. Importantly, the selective expression of mutant HTT in microglia is sufficient to increase the expression of proinflammatory genes, and to induce exaggerated neuronal death under inflammatory conditions^20^. Conversely, bone marrow transplantation of irradiated HD mice with wildtype (WT) immune cells normalises serum levels of proinflammatory factors and decreases motor dysfunction and synaptopathy^21^. Similarly, activation of cannabinoid receptor 2 signaling, which dampers immune activation, improves survival, motor, and synaptic deficits in a mouse model^22^. These studies further strengthen the hypothesis that immune dysfunction may contribute to the pathogenesis of HD.

A number of molecular pathways have been implicated in aberrant immune activation in HD, including abnormal NF-kB^23^, STAT5^24^, PU.1^20^, and P2 × 7 receptor^25^ activity. Of these, the NF-kB pathway, a key transcriptional inducer of proinflammatory factors, is hyperactive in peripheral immune and CNS glial cells (microglia and astrocytes) isolated from HD patients and mouse models^26^,^27^,^28^. This dysregulation of NF-kB signalling appears to be a direct effect of mutant HTT, mediated through its interaction with and activation of the IKK complex^23^,^26^,^28^,^29^, a positive regulator of NF-kB. Consistent with a cell-autonomous mechanism, lowering HTT levels reverses both the hyperactivation of NF-kB and the overproduction of proinflammatory factors^28^. Of potential therapeutic relevance, direct inhibition of excessive NF-kB activity normalises the levels of proinflammatory factors secreted upon activation in HD cells and, importantly, ameliorates their detrimental effects on neurons^26^. Thus, immunomodulatory agents with effects on NF-kB activity may be of therapeutic benefit in HD.

In this study, using the YAC128 mouse model of HD, we evaluated the therapeutic potential of laquinimod, a novel immunomodulatory agent currently in clinical development for multiple sclerosis. Although its precise molecular targets have not been fully defined^30^, laquinimod has been shown to act in the periphery and the CNS to reduce NF-kB activation^31^, decrease the levels of secreted proinflammatory factors, and to afford neuroprotection and improved functional outcomes in a number of neuroinflammatory conditions^30^. In addition to its modulatory effect on NF-kB-induced proinflammatory factors, laquinimod has been shown to upregulate BDNF^32^,^33^, a neurotrophic factor with reduced expression and secretion in HD^34^. These properties make laquinimod an attractive candidate for therapeutic intervention in HD. Indeed, a Phase 2 double-blind, randomized placebo-controlled, dose ranging clinical study, LEGATO-HD, is currently underway to investigate the efficacy and safety of laquinimod as a potential treatment for patients with HD.

Here, we demonstrate that laquinimod results in the rescue of striatal, cortical and corpus callosal atrophy and improvement in white matter microstructural abnormalities in YAC128 HD mice in a dose-specific manner. These data provide additional support for the concept of immune activation playing an important role in the pathogenesis of HD.

## Materials and Methods

### Animals

Male and female YAC128 HD mice (line 53) expressing a full-length human *HTT* transgene with 128 CAG repeats^35^, maintained on the FVB/N strain, were used. The mice were bred at the Biological Resource Centre (Agency for Science, Technology and Research, ASTAR). Mice were group-housed with littermates of mixed genotype. Animals were maintained under a 12 h light cycle (lights on at 09:00) in a clean facility and given free access to food and water. Experiments were performed with the approval of the Institutional Animal Care and Use Committee at the Biomedical Sciences Institute (ASTAR) and in accordance with the approved guidelines.

### Administration of laquinimod

Laquinimod was synthesized by Teva Pharmaceutical Industries and was dissolved in sterile water. Laquinimod and vehicle were administered by oral gavage daily for five days/week starting at 2 months of age, for a period of six months. Mice received vehicle (sterile water), 1 mg/kg of laquinimod, or 10 mg/kg of laquinimod at a volume of 4 mL/kg. Animals were weighed every two weeks to ensure the correct dose was maintained.

### Brain sample preparation

Mice were anaesthetised with intraperitoneal injections of ketamine (150 mg/kg)/xylazine (10 mg/kg) mixture. The animals were perfused between 9 and 9.5 months of age with ice-cold phosphate-buffered saline (PBS) followed by ice-cold 4% paraformaldehyde in PBS. Brains were removed and left in 4% paraformaldehyde for 24 h, and then stored in PBS. After weighing, the brains were transferred to a 30% sucrose solution containing 0.08% sodium azide in PBS. They were then frozen on dry ice, mounted with Tissue-TEK O.C.T. compound (Sakura, Torrance, CA, USA) and sliced coronally into 25-mm sections on a cryostat (Microm HM 500M, Richard-Allan Scientific, Kalamazoo, MI, USA). The sections were collected and stored in PBS with 0.08% sodium azide at 4 °C.

### Immunohistochemistry and stereological measurements

A series of 25-μm thick coronal sections spaced 200 μm apart spanning the striatum were stained with NeuN antibody (1:100; Chemicon) overnight at room temperature, followed by incubation with biotinylated anti-mouse antibody (1:200; Vector Laboratories, Burlingame, CA, USA). The signal was amplified with an ABC Elite kit (Vector) and detected with diaminobenzidine (Pierce). Striatal volumes were determined from a series of mounted sections using StereoInvestigator software (Microbrightfield, Williston, VT, USA) by tracing the perimeter of the striatum in serial sections spanning the striatum. Corpus callosum volumes were determined from a series of mounted sections using the Volumest plug-in for ImageJ by tracing the perimeter of the corpus callosum in serial sections spanning the striatum. A grid size of 200 μm was used for analysis. Sections incubated without primary antibody served as controls. Following a wash in PBS, sections were incubated with biotinylated donkey anti-sheep secondary antibody (1:500, Jackson ImmunoResearch Labs, West Grove, PA, USA) for 2 h in TBS-TDS. Following another wash in PBS, sections were incubated in Vectastain Elite ABC reagent (Vector Labs Inc., Burlingame, CA, USA) for 30 min. Staining was visualised using 3,3′-diaminobenzidine in 50 mM Tris-imidazole buffer (pH 7.6). After being mounted on slides, sections were dehydrated through a graded series of ethanol solutions (70, 85, 90, and 100% for 2 min each). Slides were then treated with Citrisolv (Fisher, Tustin, CA, USA) for 2 min, followed by xylene for 10 min, and cover slipped with Cytoseal 60 (Richard-Allan Scientific). Sections were photographed using a Zeiss Axioplan 2 microscope and Coolsnap HQ Digital CCD camera (Photometrics, Tucson, AZ, USA). Digital images were colour-balanced using Adobe Photoshop 7.0 (Adobe Systems, San Jose, CA, USA).

### Imaging: tensor-based morphometry (TBM)

Animals were scanned at 8 months of age on a 7T MRI scanner (ClinScan, Bruker BioSpin, Germany) using 4-channel array coils. The mice were anesthetized and maintained stably under anaesthesia by 1–2% isoflurane mixed with air and O_2_ (1:1) at a flow rate of 1L/min via a nose cone. An MRI-compatible stereotaxic holder was used to secure the head. The rectal temperature was monitored and maintained at 36 ± 0.5 C using heated air (SAII, Stony Brook, NY, USA), and the respiration rate was controlled at 100 ± 20 breaths per minute by varying the concentration of isoflurane. The structural image was acquired by a fast-spin-echo T2-weighted sequence with TR = 3080 ms, TE = 43 ms and 0.1 × 0.1 × 0.3 mm^3^ voxel resolution with coil inhomogeneity normalisation. The brain was extracted using 3D-PCNN^36^ followed by manual editing. Images were linearly registered to an in-house mouse brain template based on 8 YAC128 and 8 WT mice (9 months of age) and then averaged to create a study-specific template. Image of each subject were then non-linearly registered to the template using FSL (FMRIB Software Library v5.0, University of Oxford, Oxford, UK, http://fsl.fmrib.ox.ac.uk/). To compare voxel-wise tissue volume differences between WT and YAC128 mice, TBM was calculated based on the Jacobian determinant (a measure of volume changes from non-linear registration). Total brain volumes were quantified based on the extracted brain. A binary mask of the caudate-putamen (CPu) was manually delineated on the study-specific template, and the CPu volume for each animal was calculated based on the mean Jacobian determinant within the mask.

### Imaging: diffusion tensor imaging (DTI)

The DTI was acquired using a spin-echo Echo Planar Imaging (EPI) sequence with 9 averages of 30 diffusion sensitising directions, b = 1500 s/mm^2^, TR = 11000 ms, TE = 41 ms, voxel size = 0.25 × 0.25 × 0.3 mm^3^. Reversed phase-encoding EPI images were also collected for the purpose of distortion correction. The quality of images was checked using an in-house Matlab (Mathworks, MA, USA) code to detect and eliminate corrupted images and hyper-intensive outliers. After eddy current distortion and motion correction, susceptibility distortion in EPI was corrected by FSL TOPUP^37^,^38^. Fractional anisotropy (FA), radial diffusivity (Dr), and parallel diffusivity (Dp) were obtained by weighted least squares tensor fitting^39^. A combination of linear registration from the B0 image to the corresponding T2-weighted structural image, and the nonlinear transformation from the T2-weighted image to the above mentioned study-specific template was applied to the FA map using FSL. The registered FA maps were then averaged to create a study-specific FA template for a second round of nonlinear registration. The same transformation was applied to Dr, Dp, and MD maps. After 2D Gaussian smoothing with a kernel of 0.3 mm full width at the half maximum, voxel-wise 2-sample t-tests between groups were conducted using SPM8^40^ with a cluster threshold of p < 0.05 determined by a Monte-Carlo simulation using 3DClustSim in AFNI (NIH; http://afni.nimh.nih.gov/). Regions of interest (ROIs), including the anterior and posterior part of the corpus callosum, and cingulum, were defined on the FA template. The mean values of FA, MD, Dr, and Dp over each ROI were compared.

IL-6 ELISA. Whole blood was collected retro-orbitally after animals were anesthetized with Ketamine (150mg/Kg) and Xylazine (10 mg/Kg) via intraperitoneal injection, and before sacrifice. Blood was collected in EDTA and heparin free tubes and kept on ice until sample processing. Samples were then incubated at 37 °C for 30 min in a thermo block and centrifuged 15 min at 3000 rpm and 4 °C. Approximately between 50–100 μL of serum was collected per animal. Samples were stored at −80 °C until IL-6 levels were measured using Quantikine ELISA Mouse IL-6 immunoassay (R&D Systems, cat no. M6000B) following the manufacturer’s instructions.

### Accelerating rotarod test

The rotarod test is designed to evaluate motor coordination and balance in rodents using an accelerating rotarod apparatus (UGO Basile 47600 Rotarod, rotating rod diameter 3 cm). Training was carried out at 2 months of age, prior to the start of treatment, and consisted of three trials (120 sec each) per day spaced 1 h apart at a fixed speed of 18 rpm for three consecutive days. The testing phase was carried out every two months between 2 (baseline) and 8 months of age and consisted of three trials spaced 2 h apart where the rotarod accelerated from 5 to 40 rpm over 5 min. Rotarod scores are the average of three trials. For ease of comparison, results were normalized to baseline (pre-treatment) performance at 2 months of age.

### Climbing test

The climbing test is used to assess motor function in rodents. Each trial session consisted of an acclimatisation phase and a test phase. In the acclimatisation phase, the mice were allowed to acclimatise to the testing room for at least 30 min before testing. In the test phase, mice were placed at the bottom end of a closed-top wire mesh cylinder and their behaviour was monitored for 5 min. When a mouse’s four paws left the table top to the time when the first paw is replaced on the table top was scored as time spent climbing. The sum of climbing time for the 5 min trial is the total time spent climbing for each mouse. The latency at which each mouse started to climb was also measured.

### Porsolt forced swim test (FST) of depression

The Porsolt FST was performed as described previously^41^. Briefly, mice were placed in individual cylinders (25 cm tall × 19 cm wide) filled with room temperature water (23–25 °C) to a depth of 15 cm for a period of 6 min. The test sessions were recorded by a video camera placed directly above the cylinders. The sessions were examined blind and the last 4 min of the test session was scored using a time-sampling technique to rate the predominant behaviour over 5-sec intervals. The following behaviours were measured and recorded at the end of every 5 sec: swimming/climbing and immobility.

### Statistical analysis

Data are expressed as means ± SEM. Unless otherwise stated comparisons between treatment groups were assessed using a one-way ANOVA with Tukey *post-hoc* analysis. Where indicated, pair-wise comparisons between groups at individual time points were assessed with a Student’s t-test. Differences were considered statistically significant when p < 0.05. Information regarding sample size for the behavioural tests at each analysis point is provided in Supplementary Tables 1–5.

## Results

### Laquinimod rescues striatal atrophy and improves white matter microstructures in YAC128 mice

**Figure 1:**
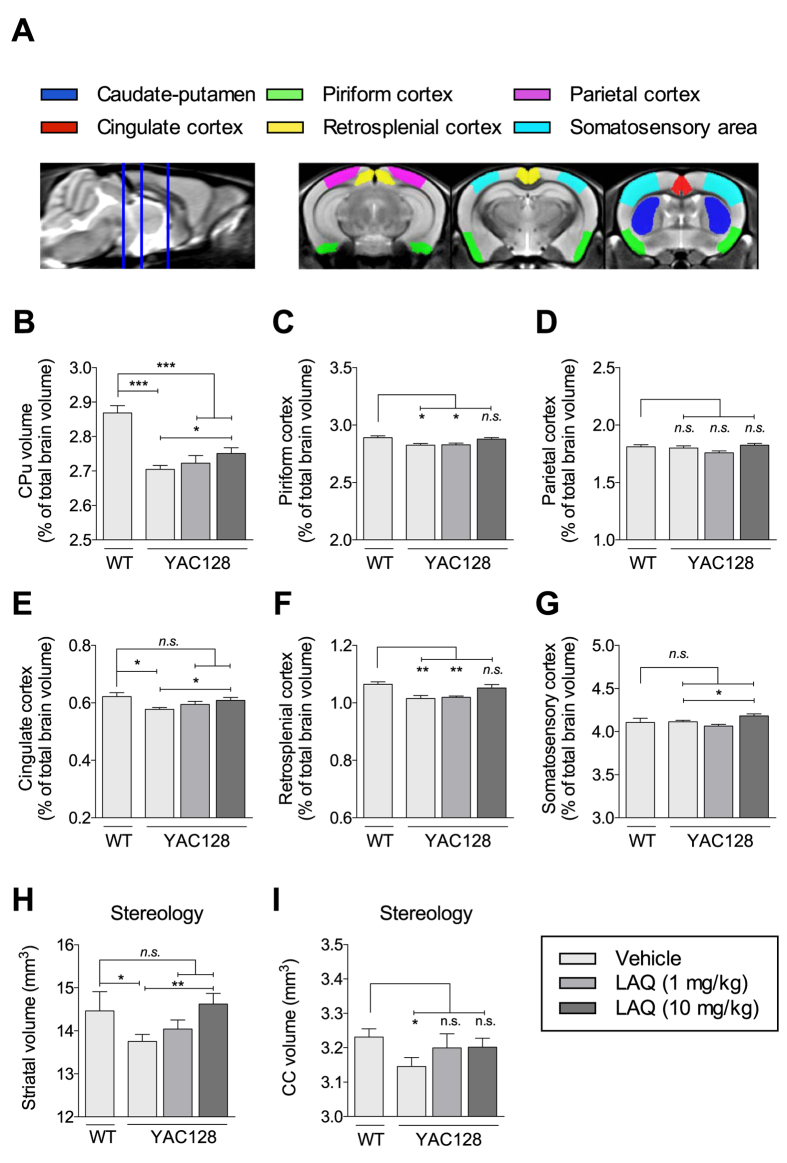
Laquinimod rescues striatal, cortical, and corpus callosum atrophy in YAC128 HD mice. Striatal and cortical atrophy was assessed by structural MRI in the caudate-putamen, and five different areas of the cortex (**A**). Vehicle-treated YAC128 mice presented striatal atrophy compared with vehicle-treated WT mice as measured by structural MRI (**B**) and stereological assessment (**H**). Treatment with the highest dose of laquinimod (10 mg/kg) rescued striatal atrophy in YAC128 mice (**A,H**). Vehicle-treated YAC128 mice displayed cortical atrophy in the piriform (**C**) cingulate (**E**) and retrosplenial (**F**) areas of the cortex as measured by structural MRI. Treatment with laquinimod rescued cortical atrophy in the piriform (**C**) cingulate (**E**) and restrosplenial (**F**) areas of the cortex in YAC128 HD mice. Laquinimod-treated (10 mg/kg) YAC128 mice increased significantly the volume of the cingulate (**E**) and somatosensory (**G**) cortical regions compared with vehicle-treated YAC128 mice. Also, vehicle-treated YAC128 HD mice presented atrophy in the corpus callosum (CC) compared with vehicle-treated WT mice as measured by stereological assessment (**I**). Both doses of Laquinimod rescued CC atrophy in YAC128 mice (**I**). (**B**) Values shown as percentage of total brain volume ± SEM; n = 9 WT-vehicle, n = 6 YAC128-vehicle, n = 8 YAC128-laquinimod (1 mg/kg), n = 8, YAC128-laquinimod (10 mg/kg); *p < 0.05, ***p < 0.001, paired two-tailed Student’s t-test. CPu – caudate-putamen. (**C,D,F**) Values shown as mean ± SEM; n = 9 WT-vehicle, n = 7 YAC128-vehicle, n = 6–8 YAC128-laquinimod (1 mg/kg), n = 8, YAC128-laquinimod (10 mg/kg); *p < 0.05, **p < 0.01, ***p < 0.001 by one-way ANOVA with Tukey’s post hoc analysis. (**E,G**) Values shown as mean ± SEM; n = 9 WT-vehicle, n = 7 YAC128-vehicle, n = 6–8 YAC128-laquinimod (1 mg/kg), n = 8, YAC128-laquinimod (10 mg/kg); *p < 0.05 by paired two-tailed Student’s t-test. (**H,I**) Values shown as mean ± SEM; n = 9–10 WT-vehicle, n = 15–16 YAC128-vehicle, n = 18–19 YAC128-laquinimod (1 mg/kg), n = 20–21 YAC128-laquinimod (10 mg/kg); *p < 0.05, **p < 0.01 by paired two-tailed Student’s t-test. LAQ – laquinimod. CC – Corpus Callosum.

Clinical symptoms in HD are paralleled by a number of neuropathological changes, including striatal and cortical atrophy and white matter (WM) abnormalities, which can be assessed using neuroimaging and stereological techniques. To assess the effect of laquinimod on HD-related neuropathology, we first measured striatal caudate-putamen (CPu) and cortical volume by structural magnetic resonance imaging (MRI) in 8-month-old mice following 6 months of treatment (Fig. 1A). Vehicle-treated YAC128 HD mice showed significantly lower striatal CPu volume compared with vehicle-treated WT mice (Fig. 1B; paired two-tailed Student’s t-test; p < 0.00001). Treatment with the highest dose of laquinimod (10 mg/kg) significantly increased the striatal CPu volume compared with vehicle-treated YAC128 HD mice (Fig. 1B; paired two-tailed Student’s t-test; p < 0.05). Stereological assessment at 9 months of age corroborated the structural MRI results, showing similar effects of laquinimod on striatal volume in YAC128 HD mice (Fig. 1H). Assessment of cortical volume was performed in five different regions of interest (ROIs): piriform, parietal, cingulate, retrosplenial, and somatosensory cortex (Fig. 1A). Vehicle-treated YAC128 mice showed significantly lower cortical volume in the piriform, cingulate, and retrosplenial areas of the cortex compared with vehicle-treated WT mice (Fig. 1C,E,F; one-way ANOVA with Tukey’s *post hoc* analysis, p < 0.05 for piriform cortex, and p < 0.01 for retrosplenial cortex; paired two-tailed Student’s t-test, p < 0.01 for cingulate cortex) whereas no difference was observed in the parietal and somatosensory area of the cortex (Fig. 1D,G; one-way ANOVA with Tukey’s *post hoc* analysis, p > 0.05 for parietal cortex; paired two-tailed Student’s t-test, p > 0.05 for somatosensory cortex). Treatment with the low (1 mg/kg) dose of laquinimod increased the volume in the cingulate area of the cortex to levels not significantly different from vehicle-treated WT mice (Fig. 1E), but no effect was observed in the other areas of the cortex (Fig. 1C,D,F,G). Treatment with the high (10 mg/kg) dose of laquinimod resulted in a significant increase in the volume of the cingulate (Fig. 1E) and somatosensory (Fig. 1G, paired two-tailed Student’s t-test; p < 0.05) cortical regions compared to vehicle-treated YAC128 mice. The volume of the piriform, cingulate and retrosplenial cortical regions in YAC128 HD mice treated with the highest dose of laquinimod was not different from that of vehicle-treated WT mice (Fig. 1C,E,F).

**Figure 2:**
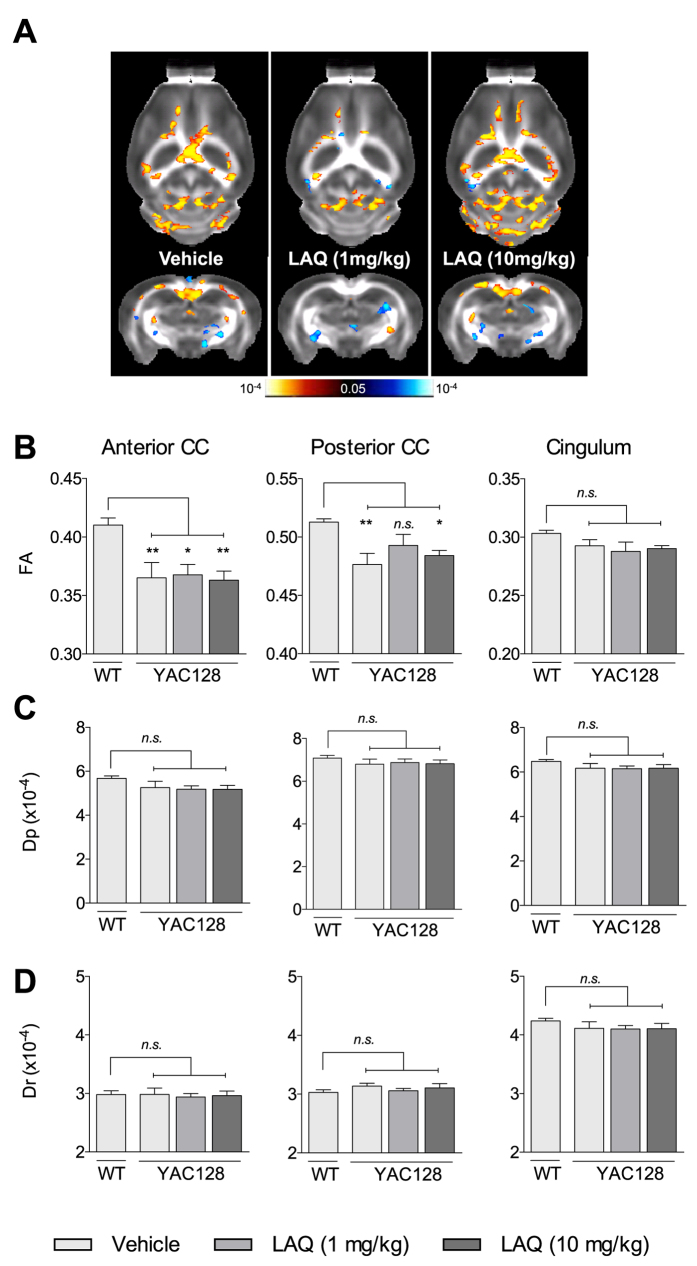
Laquinimod improves white matter microstructures in YAC128 HD mice. Analysis of white matter microstructural changes by diffusion tension imaging (DTI) revealed a regionally dependent decrease of FA values in vehicle/laquinimod-treated YAC128 mice compared with vehicle-treated WT mice (**A,B**) (red-yellow indicates higher FA values in vehicle-treated WT mice; blue-light blue indicates lower FA values). Treatment with the lowest dose of laquinimod (1 mg/kg) increased FA values in the posterior CC (**B** middle panel) but not in the anterior CC or cingulum (**B** right and left panel, respectively). No effects on FA values were observed using high dose laquinimod (10 mg/kg) (**B**). Parallel (Dp) and radial (Dr) diffusivity were also investigated but no differences were observed between vehicle- or laquinimod-treated YAC128 HD mice compared to vehicle-treated WT mice (**C,D**). (**B–D**) Values shown as mean ± SEM; n = 8 WT-vehicle, n = 7 YAC128-vehicle, n = 8 YAC128-laquinimod (1 mg/kg), n = 8, YAC128-laquinimod (10 mg/kg); *p < 0.05, **p < 0.01 by one-way ANOVA with Tukey’s post hoc analysis; CC – Corpus Callosum. LAQ – laquinimod.

YAC128 HD mice exhibit age-dependent decreases in corpus callosum (CC) volume^42^. In addition, we have recently shown that, similar to patients with HD, YAC128 HD mice present microstructural WM abnormalities especially in the CC and the cingulum^43^. To evaluate the effect of laquinimod on the integrity of WM microstructures in YAC128 HD mice, we performed magnetic resonance diffusion tensor imaging (MR-DTI) in 8-month-old mice. Fractional anisotropy (FA), a measure of the directionality of water diffusion, was investigated in three different ROIs: the anterior corpus callosum (CC), posterior CC, and the cingulum (Fig. 2). To identify WM change in the whole brain, voxel-wise analysis indicated a small but significant reduction in FA values in vehicle-treated YAC128 HD mice in the anterior and posterior CC compared with vehicle-treated WT mice (Fig. 2A,B middle panel; one-way ANOVA with Tukey’s *post hoc* analysis; p > 0.05 for cingulum, p < 0.01 for anterior and posterior CC), suggesting a loss of integrity in WM microstructures. The lowest dose of laquinimod (1 mg/kg) modestly increased FA values in the posterior CC region in YAC128 HD mice to levels not significantly different from vehicle-treated WT animals (Fig. 2B middle panel; one-way ANOVA with Tukey’s *post hoc* analysis; p > 0.05), however no effect with the highest dose was observed (Fig. 2B middle panel; one-way ANOVA with Tukey’s *post hoc* analysis; p > 0.05). Given the effects of laquinimod treatment on FA value, parallel (Dp) and radial (Dr) diffusivity values were inspected but no differences were observed between vehicle- or laquinimod-treated YAC128 HD mice compared to vehicle-treated WT mice (Fig. 2C,D; one-way ANOVA with Tukey’s *post hoc* analysis; p > 0.05). These findings suggest that low-dose laquinimod is partially able to counteract the development of disease-associated WM microstructure abnormalities in this region. The volume of the CC was measured by stereological assessment at 9 months of age. Vehicle-treated YAC128 HD mice showed significantly reduced CC volume compared with vehicle-treated WT mice, consistent with previous reports^42^,^44^ (Fig. 1I; paired two-tailed Student’s t-test; p < 0.05). Treatment with both doses of laquinimod modestly increased CC volumes in YAC128 HD mice to levels not significantly different from vehicle-treated WT mice (Fig. 1I; paired two-tailed Student’s t-test; p > 0.05).

Forebrain weight has been previously used as a neuropathological marker for assessment in the YAC128 HD mice^35^,^45^. At nine month of age, vehicle-treated YAC128 HD mice did not show forebrain weight differences compared with vehicle-treated WT mice (Supplementary Fig. 1). Treatment with low or high dose of laquinimod did not have any effect on forebrain weight in YAC128 HD mice (Supplementary Fig. 1), suggesting no detrimental effect of laquinimod with long-term (~7 months) treatment.

### Effect of laquinimod on serum interleukin-6 levels in YAC128 HD mice

**Figure 3:**
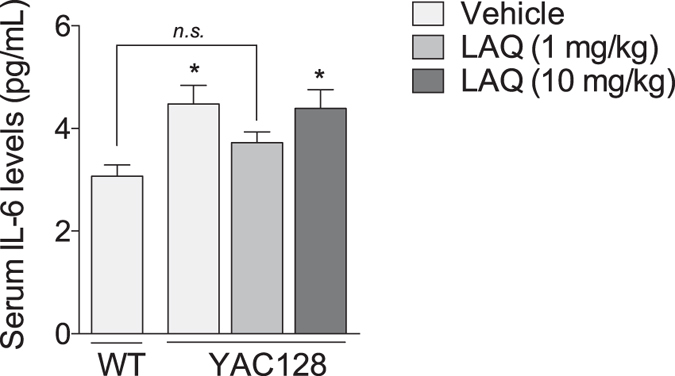
Effect of laquinimod on interleukin-6 levels in serum of YAC128 HD mice. Vehicle-treated YAC128 HD mice presented increased serum levels of interleukin-6 (IL-6) compared with vehicle-treated WT mice. Low dose laquinimod (1 mg/kg) reduced the levels of serum IL-6 in YAC128 HD mice, whereas no effect was observed with high dose laquinimod (10 mg/kg). Values shown as mean ± SEM; n = 8 WT-vehicle, n = 16 YAC128-vehicle, n = 15 YAC128-laquinimod (1 mg/kg), n = 18 YAC128-laquinimod (10 mg/kg); *p < 0.05 and n.s. = not significant (compared with vehicle-treated WT) by paired Student’s t-test. LAQ – laquinimod.

Abnormal immune activation in the CNS and periphery of HD patients and animal models, including elevated plasma levels of interleukin 6 (IL-6), has been shown previously^14^. The immunomodulatory properties of laquinimod prompted us to assess its effect on serum levels of IL-6, as a measure of peripheral immune activation. Consistent with previous findings^14^, vehicle-treated YAC128 HD mice presented increased levels of serum IL-6 compared with vehicle-treated WT mice (Fig. 3; one-way ANOVA with LSD *post hoc* analysis; p = 0.013). Serum IL-6 levels in YAC128 HD mice treated with the lowest dose of laquinimod (1 mg/kg) were reduced to levels not significantly different from those of vehicle-treated WT mice (Fig. 3; one-way ANOVA with LSD *post hoc* analysis; p = 0.243) whereas YAC128 HD mice treated with the highest dose of laquinimod (10 mg/kg) presented increased levels of serum IL-6 compared with vehicle-treated WT mice (Fig. 3; one-way ANOVA with LSD *post hoc* analysis; p < 0.0175). These results suggest that laquinimod, at low doses, may reduce peripheral immune activation in HD mice.

### Effect of laquinimod on behavioural phenotypes in the YAC128 HD mice

The YAC128 HD mice present behavioural deficits that resemble symptoms observed in patients with HD^35^,^41^,^46^. These motor, cognitive, and affective phenotypes can be used to assess the therapeutic potential of candidate therapies for HD^44^,^47^,^48^,^49^.

**Figure 4:**
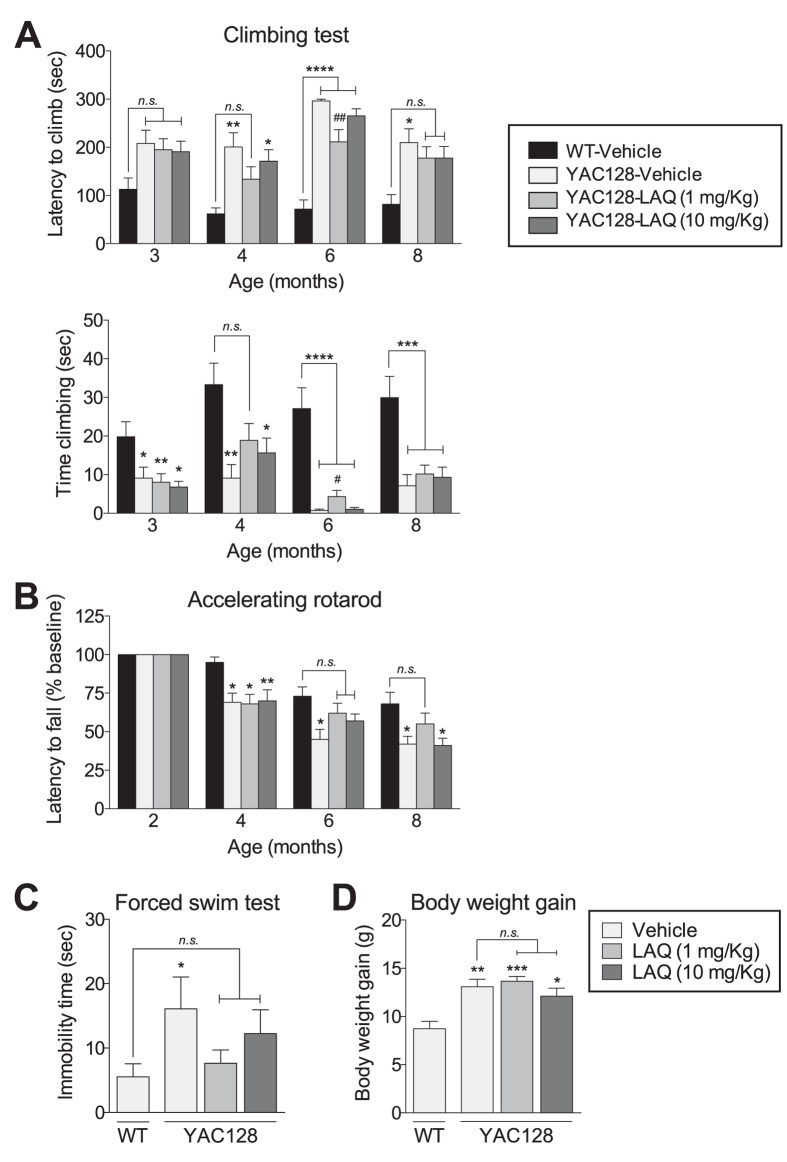
Laquinimod improves motor function and depressive behaviour in YAC128 HD mice. Vehicle-treated YAC128 HD mice displayed motor deficits in the climbing test (**A**). Low dose laquinimod partially restored performance in the climbing test in YAC128 HD mice by decreasing latency to climb at 4 and 8 months of age **(A**, upper panel) and increasing time spent climbing at 4 months of age (A, lower panel). High dose laquinimod improved latency to climb at 8 months of age **(A**, upper panel). In addition, vehicle-treated YAC128 HD mice displayed motor deficits in the accelerating rotarod test (**B**). Both doses of Laquinimod improved motor function in YAC128 HD mice at 6 months of age, which were maintained at 8 months with low dose laquinimod treatment (**B**). Also, vehicle-treated YAC128 HD mice exhibited increased immobility in the forced swim test of depression, which was reduced in laquinimod-treated YAC128 HD mice (**C**). Laquinimod treatment had no effect on weight gain in YAC128 HD mice (**D**). (**A**) Values shown as mean ± SEM; n = 10–11 WT-vehicle, n = 12–16 YAC128-vehicle, n = 19 YAC128-laquinimod (1 mg/kg), n = 19–22 YAC128-laquinimod (10 mg/kg); *p < 0.05, **p < 0.01, ****p < 0.0001 (compared with vehicle-treated WT) and ^#^p < 0.05, ^##^p < 0.01 (compared to vehicle-treated YAC128) by one-way ANOVA with LSD post hoc analysis. LAQ – laquinimod. (**B**) Values shown as percentage of baseline ± SEM.; n = 10–12 WT-vehicle, n = 16 YAC128-vehicle, n = 19 YAC128-laquinimod (1 mg/kg), n = 21–22 YAC128-laquinimod (10 mg/kg); *p < 0.05, **p < 0.01 (compared with vehicle-treated WT) by one-way ANOVA with Tukey’s post hoc analysis. (**C**) Values shown as mean ± SEM; n = 9 WT-vehicle, n = 17 YAC128-vehicle, n = 19 YAC128-laquinimod (1 mg/kg), n = 22 YAC128-laquinimod (10 mg/kg); *p < 0.05 (compared with vehicle-treated WT) by paired Student’s t-test. (**D**) Values shown as mean ± SEM; n = 12 WT-vehicle, n = 15 YAC128-vehicle, n = 19 YAC128-laquinimod (1 mg/kg), n = 22 YAC128-laquinimod (10 mg/kg); *p < 0.05, **p < 0.01, ***p < 0.001 by one-way ANOVA with Tukey’s post hoc analysis. LAQ – laquinimod.

To examine the effect of laquinimod on motor function, mice were tested using the accelerating rotarod and climbing tests (Fig. 4A,B). All mice were tested longitudinally starting at 2 months of age and ending at 8 months. By 4 months of age, significant differences in performance in the climbing test, as measured by the latency to climb, between vehicle-treated YAC128 mice and vehicle-treated WT mice were apparent (Fig. 4A upper panel; one-way ANOVA with Tukey’s *post hoc* analysis; p < 0.01 at 4 months, p < 0.0001 at 6 months, p < 0.05 at 8 months). Treatment with low dose laquinimod (1 mg/kg) reduced latency to climb by 15–35% to levels not significantly different from vehicle-treated WT mice at 4 and 8 months of age (Fig. 4A upper panel; one-way ANOVA with Tukey’s *post hoc* analysis; p < 0.0001 at 6 months). Less substantial reductions (15%) in latency to climb were observed with high dose laquinimod, although by 8 months, the latency to climb was again not significantly different from vehicle-treated WT mice (Fig. 4A upper panel; one-way ANOVA with Tukey’s *post hoc* analysis; p > 0.05).

In terms of time spent climbing, significant difference in performance between vehicle-treated YAC128 mice and vehicle-treated WT mice were apparent starting at 3 months of age (Fig. 4A lower panel, one-way ANOVA with Tukey’s *post hoc* analysis; p < 0.05 at 3 months, p < 0.01 at 4 months, p < 0.0001 at 6 months, and p < 0.001 at 8 months). Treatment with low dose laquinimod increased the amount of time the mice spent climbing by 42–108% relative to vehicle-treated YAC128 HD mice, and was similar to levels of vehicle-treated WT mice at 4 months of age (Fig. 4A lower panel; one-way ANOVA with Tukey’s *post hoc* analysis; p > 0.05 at 4 months of age, p < 0.0001 at 6 months and p < 0.001 at 8 months). No effect on time-spent climbing was seen in the high dose laquinimod treatment group.

Significant differences in performance in the accelerating rotarod test were also apparent between vehicle-treated YAC128 mice and vehicle-treated WT mice by 4 months of age (Fig. 4B one-way ANOVA with Tukey’s *post hoc* analysis; p < 0.05 at 4, 6, and 8 months). The rotarod performance of YAC128 mice was improved with low and high dose laquinimod treatment by 27–37% to levels not significantly different from that of vehicle-treated WT mice at 6 months of age. This improvement was maintained at 8 months of age in the low dose laquinimod-treated YAC128 mice (Fig. 4B; one-way ANOVA with Tukey’s *post hoc* analysis; p > 0.05 compared with vehicle-treated WT). The results of the climbing and accelerating rotarod tests indicate that laquinimod treatment improves motor function in YAC128 HD mice.

Next, we evaluated the effect of laquinimod on depressive-like behaviour in the Porsolt forced swim test. Vehicle-treated YAC128 mice displayed depressive-like behaviour (increased immobility) compared with vehicle-treated WT mice, consistent with previous findings (Fig. 4C; paired t-test; p < 0.05)^41^,^44^,^45^,^50^. The depressive-like behaviour (immobility time) of YAC128 HD mice treated with either low or high dose of laquinimod was reduced by 30–60% to levels not significantly different from those of vehicle-treated WT mice. (Figure 4C; paired t-test; p > 0.05 compared with vehicle-treated WT). This finding suggests that laquinimod treatment improves the depressive-like phenotype of YAC128 HD mice.

No differences between vehicle-treated WT and YAC128 mice were observed in the spontaneous alternation and the novel object recognition/location tests of cognition, and in the elevated plus maze and open field tests of anxiety (data not shown). As such, we were not able to assess the potential for laquinimod to improve cognitive function or anxiety-like behaviour in YAC128 HD mice.

### Effect of long-term laquinimod treatment on weight gain and survival

To address the possibility that long-term laquinimod treatment may be detrimental, body weight gain and survival rate were compared across the groups at the end of the treatment period (Fig. 4D). Consistent with the previous findings^51^,^52^, we observed a greater increase in body weight in YAC128 HD mice compared with WT mice (Fig. 4D, one-way ANOVA with Tukey’s *post hoc* analysis; p < 0.01). No differences in body weight gain between laquinimod- and vehicle-treated YAC128 HD mice were observed (Fig. 4D; one-way ANOVA with Tukey’s *post hoc* analysis; p > 0.05). Finally, survival analysis was performed and no differences were observed across all the groups (Supplementary Fig. 2). Altogether, these results suggest that long-term laquinimod treatment is well tolerated and does not cause detrimental effects in YAC128 HD mice.

## Discussion

Accumulating evidence has implicated immune dysfunction, and in particular hyperactivity of NF-kB signalling, in the pathogenesis of HD, suggesting that interventions targeting this pathway may be of therapeutic value. Here we show that laquinimod, a brain-penetrant immunomodulatory agent previously shown to mitigate excessive NF-kB activation in the periphery and CNS^31^, improves neuropathology and some behavioural deficits in the YAC128 HD mice. Treatment with laquinimod for 6 months rescued atrophy in the striatum, in certain cortical regions, and in the corpus callosum of YAC128 HD mice. Diffusion tensor imaging showed that white matter microstructural abnormalities in the posterior corpus callosum of YAC128 HD mice were also improved following laquinimod treatment, and were paralleled by reduced levels of the immune factor IL-6 in the periphery. Functionally, laquinimod treatment led to modest improvements in motor function, as measured by the climbing and accelerating rotarod tests, and in depressive-like behaviour measured using the forced swim test. Taken together, these results suggest that laquinimod may confer functional benefits in HD.

LEGATO-HD, a Phase 2 double-blind, randomized placebo-controlled, dose ranging clinical trial of laquinimod as a potential treatment for patients with HD, is currently underway. LEGATO-HD’s design utilizes a combination of well-established and exploratory endpoints, soluble biomarkers and imaging methodologies that are expected to advance the understanding of the role of inflammation and immunomodulation in HD patients and translate the preclinical observations in this report. MRI measures of whole brain volume, caudate volume, white matter and ventricular volume are collected after 12 months of treatment. In addition in 4 ancillary studies PET and MRS Imaging is conducted, and biological material is collected for immune and proteomic analysis at selected study sites.

While this paper was under review, a study was published in which the effect of laquinimod on myeloid cells of patients with HD was evaluated *ex vivo*^53^. Monocytes of premanifest and manifest HD gene carriers that were pre-treated with laquinimod for 24 hr *ex vivo* released lower levels of inflammatory factors following stimulation compared with monocytes of healthy volunteers^53^, supporting a dampening effect of laquinimod on the hyperactive inflammatory myeloid response in HD.

Preclinically, laquinimod has shown protective effects in a number of models of neuroinflammation^30^. These include experimental autoimmune encephalomyelitis models of MS^33^,^54^,^55^,^56^,^57^,^58^, the cuprizone model of acute demyelination^31^, the experimental autoimmune neuritis model of Guillain-Barré syndrome^59^,^60^, and a rodent model of systemic lupus erythematosus^61^. Its immunomodulatory effects have been shown to reflect, at least in part, moderation of NF-kB signalling^31^. Thus, modulation of HD-related pathological immune activation, possibly through NF-kB modulation, may have contributed to the improved neuropathology and functional outcomes reported here.

In terms of dosage effects, the majority of measures assessed did not show dose-dependence. This is consistent with a number of previous studies in which dose-dependence for laquinimod was not observed^31^,^59^,^62^. Nonetheless, some distinct effects of the two doses evaluated were observed. Whereas both doses restored striatal volume of YAC128 HD mice to WT levels, only the low dose (1 mg/kg) improved WM microstructural abnormalities and serum IL-6 levels. Furthermore, the improvements in motor function appeared earlier and persisted longer in the low dose-treated group. These observations suggest that multiple, dose-sensitive mechanisms might be mediating the action of laquinimod.

Indeed, in addition to immunomodulation, laquinimod has been shown to enhance expression of the neurotrophic factor BDNF in a dose-specific manner^32^,^33^,^62^. Deficits in BDNF expression, transport, and action are well documented in cellular and animal models of HD, including YAC128 HD mice^34^,^49^,^50^. Though not examined in the present study, the improvements we observed in specific aspects of neuropathology and behaviour might be a consequence of restored BDNF expression following laquinimod treatment in YAC128 mice. Of note, a recent study has shown that the laquinimod-induced increase in BDNF expression is accompanied by anti-depressive effects in the forced swim test of depression^62^, a finding consistent with the improved depressive behaviour we observed in the YAC128 HD mice. Further dose-control experiments may be required to optimise the therapeutic value of laquinimod for HD.

The white matter microstructural abnormalities we observed in YAC128 HD mice, signified by reduced FA values, are in agreement with our previous findings^43^. Indeed, we have recently shown that YAC128 HD mice exhibit structural and molecular myelination deficits, which are paralleled by reduced FA values in a number of white matter-rich regions. These white matter microstructural deficits are thought to reflect, at least partly, cell-intrinsic effects of mutant HTT in oligodendrocytes^43^. The relationship between such changes in white matter integrity and HD symptoms is becoming increasingly recognised. In a recent longitudinal study examining white matter abnormalities in HD gene carriers and patients and their relationship to disease symptoms, decreased FA values in the splenium (posterior) of the corpus callosum showed a significant correlation with total motor scores on the Unified Huntington’s Disease Rating Scale^63^. Similarly, reduced FA values in the splenium were significantly correlated with depression scores for gene carriers who were closest to onset^64^. Interestingly, in this study we found that increased FA values in the posterior corpus callosum (splenium) were significantly correlated with higher motor performance (climbing time) (data not shown).

A recent study has provided evidence for a causal role for glia in HD^65^. Human HD glia derived from human HD embryonic stem cells were shown to impart disease phenotypes when engrafted in normal mice^65^. Conversely, normal human glia derived from control human embryonic stem cells ameliorated behavioural and electrophysiological disease manifestations when engrafted in HD mice, suggesting a causal role for glia in HD^65^. The cellular target for laquinimod is glia, and in particular astrocytes and microglia. These data further support targeting these cell types for therapeutic development in HD.

A caveat of the DTI analysis in this study is the possibility that the parallel and radial diffusivity measures might be biased by the free water diffusivity especially for regions close to the ventricles. Since no significant ventricular volume changes were observed in the tensor-based morphometry analysis across the groups and a high b-value of 1500 s/mm^2^ was used, the impact on the group difference may be negligible^66^. A further drawback of the present study is that the effects of laquinimod on HD-related cognitive dysfunction and anxiety phenotypes could not be evaluated. While select behavioural tests examining these aspects of disease were attempted, no deficits in the YAC128 control group relative to WT were detected, thus precluding the possibility of examining potential restorative effects of laquinimod in these domains. Furthermore, treatment in this study was initiated at 2 months of age, prior to onset of overt disease phenotypes which first appear at 3 months of age^45^. Thus it is interesting to speculate whether treatment with laquinimod either earlier or in advanced stages of disease would result in greater or fewer beneficial effects, respectively.

Overall, the effects we observed, while positive, were modest. This is consistent with previous studies targeting the immune system where partial improvements in disease phenotypes were observed^21^,^22^. In recent years, a number of HD-related pathogenic pathways with potential for therapeutic intervention have been identified. These include synaptic dysfunction^47^,^48^,^67^, kynurenine pathway hyperactivity^68^, sigma-1 receptor^69^,^70^ and cAMP/cGMP signalling^71^, and abnormal monoamine oxidase activity^72^,^73^. Given the multiplicity of pathogenic pathways, targeting multiple non-overlapping pathways in the form of combination therapy is likely to yield greater benefit than monotherapy.

## Additional Information

**How to cite this article**: Garcia-Miralles, M. *et al*. Laquinimod rescues striatal, cortical and white matter pathology and results in modest behavioural improvements in the YAC128 model of Huntington disease. *Sci. Rep.* **6**, 31652; doi: 10.1038/srep31652 (2016).

## Supplementary Material

Supplementary Information
