# Supplementary material for: Laquinimod rescues striatal, cortical and white matter pathology and results in modest behavioural improvements in the YAC128 model of Huntington disease
Source: Sci Rep. 2016 Aug 16;6:31652. doi: 10.1038/srep31652 (PMC4985819; doi:10.1038/srep31652)
Supplement: Supplementary Information [file srep31652-s1.pdf]

Laquinimod rescues striatal, cortical and white matter pathology and results in modest behavioural improvements in the YAC128 model of Huntington disease

Marta Garcia-Miralles, Xin Hong, Liang Juin Tan, Nicholas S. Caron, Yihui Huang, Xuan Vinh To, Rachel Yanping Lin, Sonia Franciosi, Spyros Papapetropoulos, Liat Hayardeny, Michael R. Hayden, Kai-Hsiang Chuang, Mahmoud A. Pouladi

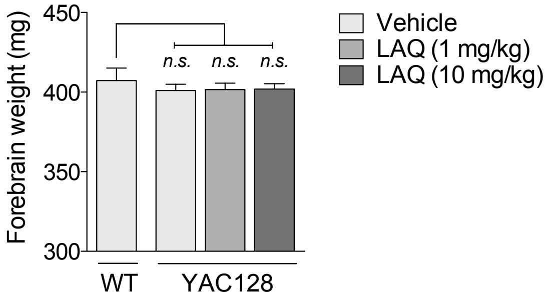

Supplementary Figure 1. Forebrain weight of vehicle- and laquinimod-treated YAC128 HD mice.

The forebrain weight of vehicle-treated YAC128 HD mice did not differ from that of vehicle-treated WT mice. Treatment with laquinimod had no effect on forebrain weight in YAC128 HD mice. Values shown as mean. Error bars represent the standard error of the mean; n = 9 WT-vehicle, n = 15 YAC128-vehicle, n = 16 YAC128-laquinimod (1 mg/kg), n = 20 YAC128-laquinimod (10 mg/kg); LAQ – laquinimod.

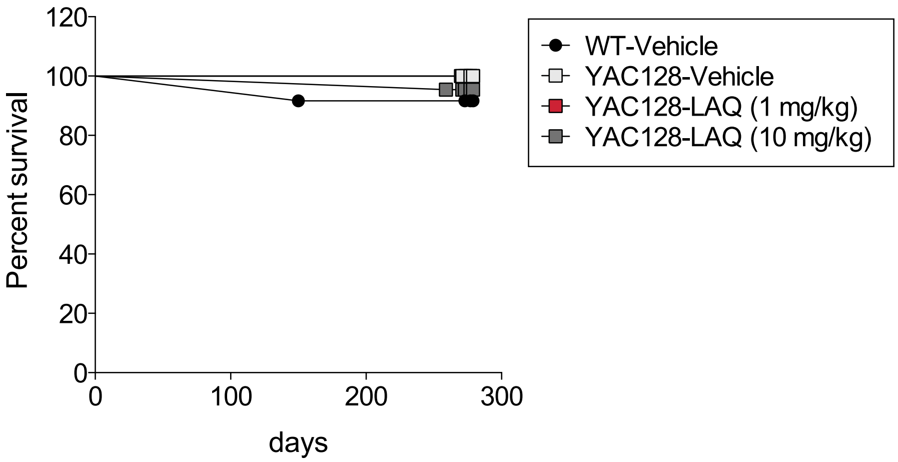

Supplementary Figure 2. Survival analysis for vehicle- and laquinimod-treated YAC128 and vehicle-treated WT mice.

Survival analysis reveals no differences between vehicle-treated YAC128 and WT mice, and no effect of laquinimod treatment on survival in YAC128 HD mice.

Supplementary Table 1. Accelerating rotarod sample size at each analysis point.

		Accelerating rotarod sample size											
		2 months			4 months			6 months			8 months		
Genotype	Treatment	F	M	N	F	M	N	F	M	N	F	M	N
WT	Vehicle	6	6	12	6	6	12	5	5	10	5	5	10
YAC128	Vehicle	8	8	16	8	8	16	8	8	16	8	8	16
YAC128	LAQ (1 mg/kg)	11	8	19	11	8	19	11	8	19	11	8	18
YAC128	LAQ (10 mg/kg)	13	9	22	13	9	22	13	9	22	13	8	21

Supplementary Table 2. Climbing test (time climbing) sample size at each analysis point.

		Climbing test sample size (time climbing)											
		3 months			4 months			6 months			8 months		
Genotype	Treatment	F	M	N	F	M	N	F	M	N	F	M	N
WT	Vehicle	5	6	11	6	6	12	5	6	11	5	5	10
YAC128	Vehicle	5	8	13	8	6	14	7	7	14	7	8	15
YAC128	LAQ (1 mg/kg)	10	8	18	11	8	19	9	8	17	10	8	18
YAC128	LAQ (10 mg/kg)	12	9	21	13	9	22	9	9	17	12	8	21

Supplementary Table 3. Climbing test (latency to climb) sample size at each analysis point.

		Climbing test sample size (latency to climb)											
		3 months			4 months			6 months			8 months		
Genotype	Treatment	F	M	N	F	M	N	F	M	N	F	M	N
WT	Vehicle	5	5	10	6	5	11	6	5	11	5	5	10
YAC128	Vehicle	5	8	13	8	6	14	6	6	12	8	8	16
YAC128	LAQ (1 mg/kg)	11	8	19	11	8	19	11	8	19	11	8	19
YAC128	LAQ (10 mg/kg)	13	9	22	13	9	22	10	9	19	13	9	22

Supplementary Table 4. Forced swim test sample size at 8 months of age.

		Forced swim test sample size		
		8 months		
Genotype	Treatment	F	M	N
WT	Vehicle	5	5	10
YAC128	Vehicle	8	7	15
YAC128	LAQ (1 mg/kg)	10	7	17
YAC128	LAQ (10 mg/kg)	11	9	20
